# Genetic influence on choroidal vascularity index

**DOI:** 10.1371/journal.pone.0318369

**Published:** 2025-01-30

**Authors:** Je Moon Yoon, Yong Jin Gil, Joohon Sung, Don-Il Ham, Mingui Kong

**Affiliations:** 1 Department of Ophthalmology, Samsung Medical Center, Sungkyunkwan University School of Medicine, Seoul, Republic of Korea; 2 Division of Genome and Health Big Data, Department of Public Health Sciences, Graduate School of Public Health, Seoul National University, Seoul, Republic of Korea; 3 Department of Ophthalmology, Kangbuk Samsung Hospital, Sungkyunkwan University School of Medicine, Seoul, Republic of Korea; Yokohama City University, JAPAN

## Abstract

**Purpose:**

To investigate the heritability of genetic influence on macular choroidal vascularity index (CVI).

**Methods:**

Total choroidal area (TCA), luminal area (LA), and CVI was measured using horizontal scan of spectral-domain optical coherence tomography with enhanced depth imaging in the 373 healthy twin participants. Characteristics of the participants were investigated, including age, sex, axial length, hypertension, diabetes, drinking habits, and smoking status. Univariate and subsequent multivariate regression analyses were performed to evaluate the associations of these factors with TCA, LA, and CVI.

**Results:**

Patients who were older and had a higher intraocular pressure, higher diastolic blood pressure, and lower pulse pressure showed associations with lower CVI (p<0.001, p = 0.014, p = 0.005, and p = 0.015, respectively). The covariate-adjusted heritability (±standard error) of the CVI was 0.716 ± 0.091, and the heritabilities of the TCA and LA were 0.691 ± 0.089 and 0.634 ± 0.100, respectively.

**Conclusion:**

The TCA, LA, and CVI are highly heritable.

## Introduction

The genetic influence on diseases, including age-related macular degeneration (AMD), primary open-angle glaucoma, or myopia, has been vigorously investigated [1–3]. These genetic components have provided a better insight of the underlying pathogenesis of major ocular diseases. Besides, several ocular traits including ganglion cell complex thickness, refractive error, or axial length (AXL), which are directly linked or contributes to some specific ocular disorder like glaucoma or myopia, also have the genetic influences [4, 5]. Discovering the genetic foundation of certain ophthalmic characteristics could promote the identifications of specific genes involved in related ophthalmic disorders.

Choroid is an important region of the eye that is associated with several macular diseases, including AMD, pachychoroid spectrum diseases, and reticular pseudodrusen [6–8]. Moreover, disease expression in nonexudative AMD varies with choroidal thickness, one of the phenotypes of the choroid [9]. The subfoveal choroidal thickness is modestly heritable, with an estimated heritability of 0.40, according to a study on Amish twins [10]. Furthermore, High heritability of choroidal volume (0.76) was revealed by the Korean Healthy Twin Study, which may improve the quantitative assessment of the amount of choroidal tissue [11, 12].

However, considering the distinguishing feature of choroid among tissues in the eye, the vascularity of the choroid should be more significant in the pathogenesis of various eye diseases. Choroidal vascularity index (CVI) is another phenotype of the choroid, representing the status of choroidal vascular component, and used to study vascular changes of the choroid in normal and various disease states [13–18]. Furthermore, while choroidal thickness was affected by many physiological factors and systemic factors, CVI was not affected by most of the variables [13]. Therefore, CVI may be a proper phenotype to measure the degree of genetic influence of the choroid; however, no study has yet explored it. In this study, we investigated the heritability of CVI in the Korean population through the Healthy Twin Study.

## Methods

### Setting

Cross-sectional data from the Healthy Twin Research were utilized in this study. Participant recruitment occurred between September 15, 2005 and December 31, 2013. In order to look into the genetic and environmental influences on a variety of characteristics, it enlists Korean adult twins and their extended families. A more detailed description of the methodology and protocols of the Healthy Twin Study has been published previously [19, 20]. Briefly, The Healthy Twin Study is a nationwide prospective cohort study that recruited Korean adult twins and their family members to investigate genetic and environmental determinants of various traits. The study recruited adult like-sex twins over age 30 and their first-degree family members who were over age 20. A family unit consisted of either twin pairs only or twin pairs with their family members (≥4 persons in a family, including parents, siblings, offspring and spouses). Participants were recruited through advertisement in nationwide newspapers, posters in hospitals and health-related governmental agencies, and direct mail contact to registered twins. The study protocol included comprehensive health examinations, questionnaires, and collection of biological specimens at one of three clinical centers (Seoul, Pusan, and Cheonan). Standard protocols and training of research coordinators/assistants were implemented across centers to ensure consistency in data collection. Also, the methodology and protocols of both ocular and systemic variables are same as previous studies [11, 21–23]. The institutional review board at Samsung Medical Center gave its approval to this study (IRB file number 2005-08-113), and it strictly followed by the principles of the Helsinki Declaration. All participants gave their written consent after being informed about the study’s purpose and potential outcomes.

### Participants

Among the entire cohort (649 participants) in the Healthy Twin Research, 418 participants had completed an optical coherence tomography (OCT) scan at the Samsung Medical Center from Mar 16, 2012 to Dec 15, 2012. Forty-five participants who exhibited pathologies potentially impacting the choroid or presented suboptimal image quality were systematically excluded from the entire cohort. The following list of factors led to exclusion: 30 had high myopia (> 6.0 diopter); 1 had retinal pigment epithelium atrophy; 4 had AMD; 3 had epiretinal membrane; and 7 had having artifacts which make difficult to describe the choroidal boundary or poor image quality (quality score < 20 dB). Finally, the study included 373 participants from 79 families, with an average of 4.61 family members and an extra 9 family members who were included as individuals. This investigation contains 54 monozygotic (MZ) twin pairs, 6 dizygotic (DZ) twin pairs, 272 sibling pairs, 94 father-offspring pairs, and 114 mother-offspring pairs.

### Choroidal vascularity index measurement

A spectral domain-OCT (Spectralis OCT; Heidelberg Engineering, Heidelberg, Germany) with enhanced depth imaging (EDI SD-OCT) was used to obtain OCT scans. One expert technician performed all of the OCT scans. The raster scan image consisted of 31 B-scans that were each composed of 768 A-scans, each measuring 9.0 mm in length, spaced 240 m apart, and covered a 30° by 30° region. Each b-scan was obtained as an average of 25 frames.

Evaluation of the total choroidal area (TCA), luminal area (LA), and CVI by binarization technique was performed as reported [13]. To assess CVI, the raw EDI SD-OCT B-scans centered at the fovea were imported into ImageJ (National Institutes of Health, Bethesda, MD). A Niblack’s autolocal threshold tool was used for the binarization of the OCT image, as reported. Manual segmentation of the choroid was performed using the “polygon tool” in ImageJ software (the nasal margin was the edge of the optic nerve head and the temporal margin was 7500 μm temporal from the edge of the optic nerve head). TCA and the area of the dark pixels were calculated. The LA was defined as the area of dark pixels. Stromal area was further calculated by subtracting LA from TCA. To determine the vascularity status of the choroid, CVI was computed by dividing LA by TCA.

### Analysis of clinical data, ocular parameters and fundus color photographs

All study subjects have received a comprehensive ophthalmic examination. We performed visual acuity assessment, intraocular pressure (IOP) measurement, non-dilated refraction measurement with an autorefractor (Topcon AT; Topcon Corporation, Tokyo, Japan), and AXL measurement using an A-scan ultrasonography (model 820; Allergan-Humphrey, San Leandro, California, USA). To detect any retinal pathology, fundus color photography was performed using the TRC 50 (Topcon, Paramus, New Jersey, USA) or Nonmyd 7 (Kowa, Tokyo, Japan) during pupil dilatation. The clinical information, ocular characteristics, and color fundus photographs were evaluated by two retinal experts (J.Y. and M.K.) to evaluate amblyopia, a history of ophthalmic surgery, and retinal or choroidal disorders that might influence a choroid.

### Evaluation of systemic variables

Routine blood pressure (BP) measurements and blood tests were performed in all study subjects. Using a standard mercury sphygmomanometer, a qualified research nurse measured the blood pressure of subjects twice, and the mean of two readings was used for analysis. Using the ADVIA 1650 analyzer and enzymatic or homogeneous assay packages, the concentrations of glucose, hemoglobin A1c, high-density lipoprotein cholesterol, low-density lipoprotein cholesterol, and triglycerides were determined in fresh serum that was collected after a minimum 12-hour overnight fast (Siemens, Munich, Germany). High systolic (>140 mm Hg), high diastolic (>90 mm Hg), or current usage of a BP lowering medication were all considered to have hypertension. A fasting glucose level of greater than 126 mg/dL, a hemoglobin A1c level greater than 6.5%, or usage of a glucose-lowering medication were all considered to have diabetes. Using a self-administered questionnaire, information about medical history, smoking status, and drinking patterns was collected.

### Statistical analysis

TCA, LA, and CVI from the right eye of every participants were used in the analyses. For each analysis, a different number of participants is used to account for missing values and family structures. The analyses of demographic and clinical characteristics in the participants were performed. As an initial analysis, multivariate linear regression was used to evaluate the associations of the CVI with each of other factors (ocular and systemic) considering age as a covariate to account for the strong impact of age on the phenotype of interest. The subsequent multiple linear regression analyses included parameters presenting an association (p<0.10) in the initial step of regression analysis.

The heritability of CVI was calculated using variance-component methods [24]. Methodologies in calculation of heritability are described in previous studies [11, 21–23]. In the present study, narrow-sense heritability was calculated to evaluate the genetic component of the choroidal vascular index and its components and the TCA and the LA. By measuring the percentage of a feature’s variance that can be accounted for by a combination of genetic factors, such as additive, dominant genetic influences, and epistasis, the variance-component technique assesses broad-sense heritability. Conversely, a narrow-sense heritability is the proportion of variance in a feature due to the additive genetic effects only. The total phenotypic variance (p^2^) of CVI was separated into additive genetic components (a^2^), shared environmental components (c^2^), and individual-specific unique environmental components (e^2^). Assumptions of the present study models are that the effects of shared environmental factors (c^2) are common among family members and that the three factors (a^2^, c^2^, and e^2^) are independent on the variance of the trait and to each other. Therefore, the total phenotypic variance equals the sum of the comprising variance components (p^2^ = a^2^+c^2^+e^2^). We fitted all plausible models, including ACE, AE, CE, or E and selected the best-fitting model with Akaike’s information criterion value. The narrow-sense heritability (h^2^) was calculated as the proportion of the additive genetic components to the total variance (a^2^/p^2^). We adjusted for age, sex, AXL, IOP, diastolic BP, pulse pressure, total cholesterol, diabetes, and hypertension in the linear mixed model for estimation of heritability, as these traits may influence CVI. Descriptive statistics, regression analysis was performed using R version 4.2. The Sequential Oligogenic Linkage Analysis Routines Eclipse, version 8.4.2 (Southwest Foundation for Biomedical Research, San Antonio, Texas, USA), was used for heritability analyses.

## Results

Among participants, 142 were male and 231 were female. The average age of 48.3 ± 13.6 years (range, from 18 to 80 years). In total, 94 subjects (28.1%) had hypertension and 22 (6.6%) had diabetes. Within the cohort, none of the patients had undergone earlier vitrectomy, buckling, or glaucoma surgery, and only three eyes had undergone cataract surgery in the past. Table 1 shows the characteristics of the participants.

**Table 1:** The demographic and clinical characteristics of the study participants.

Characteristics	Value
Age, years	48.3 ± 13.6 (18–80)
Sex, male, %	142 (38.1)
Hypertension, %	94 (28.1)
Diabetes, %	22 (6.6)
Systolic BP, mmHg	117.5 ± 15.9 (85–188)
Diastolic BP, mmHg	75.2 ± 10.2 (52–110)
Pulse pressure, mmHg	42.2 ± 9.9 (22–98)
Fasting blood sugar, mg/dL	90.4 ± 11.5 (56–165)
HDL-C, mg/dL	48.1 ± 10.6 (23–81)
LDL-C, mg/dL	110.8 ± 29.1 (38–217)
Total cholesterol, mg/dL	192.8 ± 34.8 (98.88–320)
TG, mg/dL	110.8 ± 68.6 (26–539)
ApoA1, mg/dL	150.6 ± 25.5 (95–230)
ApoB, mg/dL	92.5 ± 27.0 (41–201)
Mean pulsewave velocity, m/s	6.47 ± 1.05 (4.1–10.3)
Alcohol consumption	
None, %	144 (38.6)
Once a week or less	155 (41.6)
More than once a week	74 (19.8)
Smoking history	
Never	255 (68.4)
More than 20 packs	118 (31.6)

ApoA, apolipoprotein A; ApoB, apolipoprotein B; BP, blood pressure; HDL-C, high-density lipoprotein cholesterol; LDL-C, low-density lipoprotein cholesterol; TG, triglyceride

Continuous variables are described as mean ± standard deviation (range), and categorical parameters are described as total numbers (percentage)

Table 2 represents ocular parameters of the study participants. The AXL was 23.66 ± 1.02 mm (range: 20.86–25.99) and the spherical equivalent was −0.46 ± 1.54 diopters (range: -6–5.5). The TCA was 2.74 ± 0.86 mm^2^ (range: 0.98–5.37) and the LA was 1.81 ± 0.53 mm^2^ (range: 0.65–3.45). The CVI was 66.48 ± 2.83% (range: 57.38–75.59).

**Table 2:** Ocular parameters of the study participants.

Parameters	Value
Axial length, mm	23.66 ± 1.02 (20.86–25.99)
IOP, mmHg	14.42 ± 2.80 (8.8–24.20)
Spherical equivalent, diopter	−0.46 ± 1.54 (−6–5.5)
UDVA, logMAR	0.77 ± 0.47 (0.01–5.0)
BCVA, logMAR	1.08 ± 0.29 (0.4–1.5)
Luminal area, mm^2^	1.81 ± 0.53 (0.65–3.45)
Total choroidal area, mm^2^	2.74 ± 0.86 (0.98–5.37)
Choroidal vascularity index, %	66.48 ± 2.83 (57.38–75.59)

BCVA, best-corrected visual acuity; CVI, choroidal vascularity index; IOP, intraocular pressure; logMAR, logarithm of the minimum angle of resolution; UDVA, uncorrected visual acuity. Variables are described as mean ± standard deviation (range)

Table 3 shows the findings of the CVI regression analyses including both of univariate and multivariate. Patients who were older, had a higher IOP, higher diastolic BP, and lower pulse pressure showed associations with a lower CVI in the multivariate regression analysis with β of −0.059, −0.124, −0.042, and 0.037, respectively.

**Table 3:** Regression analyses of systemic and ocular factors associated with the choroidal vascularity index.

	Univariate*		Multivariate^†^ (N = 369)		
	Standardized beta	p value	*N*	Standardized beta	p value
Age^‡^	-0.060 ± 0.010	<0.001	373	-0.059 ± 0.012	<0.001
Sex (Male)		0.255	373		
Axial length		0.896	371		
IOP	-0.136 ± 0.050	0.007	373	-0.124 ± 0.050	0.014
Spherical equivalent		0.291	371		
UDVA		0.837	373		
BCVA		0.482	114		
Hypertension		0.202	335		
SBP		0.403	369		
DBP	-0.042 ± 0.015	0.005	369	-0.042 ± 0.015	0.005
Pulse pressure	0.025 ± 0.015	0.100	367	0.037 ± 0.015	0.015
Diabetes		0.255	335		
Fasting blood sugar		0.317	373		
Total cholesterol	-0.007 ± 0.004	0.098	373	-0.005 ± 0.004	0.204
HDL-C		0.877	373		
LDL-C		0.331	373		
TG		0.277	372		
ApoA1	-0.021 ± 0.010	0.038	104		
ApoB		0.869	104		
Mean pulsewave velocity		0.458	366		
Alcohol consumption			373		
Never	(reference)				
Equal to or less than 1/week	-0.169 ± 0.324	0.603			
More than 1/week	-0.389 ± 0.389	0.317			
Smoking history			373		
Never	(reference)				
More than 20 packs	0.025 ± 0.302	0.934			

ApoA, apolipoprotein A; ApoB, apolipoprotein B; BCVA, best-corrected visual acuity; CVI, choroidal vascularity index; DBP, diastolic blood pressure; HDL-C, high-density lipoprotein cholesterol; IOP, intraocular pressure; LDL-C, low-density lipoprotein cholesterol; SBP, systolic blood pressure; TG, triglyceride; UDVA, uncorrected visual acuity

*Adjusted for age

^**†**^Adjusted for variables with a p value <0.10 in the univariate analysis. (Without ApoA1 due to numerous missing values)

^‡^Univariate analysis of age alone

Table 4 demonstrates the heritability of TCA, LA, and CVI with adjustments for covariates, including age, gender, AXL, IOP, diastolic BP, pulse pressure, total cholesterol, diabetes, and hypertension. The covariate-adjusted heritability of the TCA, LA, and CVI were 0.691, 0.634, and 0.716, respectively.

**Table 4:** Heritability (h^2^) of the total choroidal area, luminal area, and choroidal vascularity index (N = 331).

	Variance Component*				Heritability*		Variance Explained by Covariate	Significant Covariates Included
	A	C	E	Best-fitting model	h^2^ ± SE	p value		
Total choroidal area^†^	0.691	0.000	0.309	AE	0.691 ± 0.089	<0.001	0.211	Age, sex, axial length, IOP, DBP, hypertension
Luminal area^‡^	0.634	0.000	0.366	AE	0.634 ± 0.100	<0.001	0.232	Age, sex, axial length, IOP, Hypertension
Choroidal vascularity index	0.716	0.000	0.284	AE	0.716 ± 0.091	<0.001	0.125	Age, IOP, DBP, pulse pressure, diabetes, total cholesterol

A, additive genetic components; C, shared environmental components within a family; DBP, diastolic blood pressure; E, individual-specific unique environmental components; HTN, hypertension; h2, heritability estimates; IOP, intraocular pressure

*Adjusted for age, sex, axial length, intraocular pressure, diastolic blood pressure, pulse pressure, total cholesterol, diabetes, and hypertension

^**†**^Analysis result for N = 329

^‡^Analysis result for N = 332

## Discussion

In this population-based study of Korean twins and their family members, we found that CVI is significantly influenced by genetic factors by showing high heritability after adjusting for age, sex, AXL, IOP, diastolic BP, pulse pressure, total cholesterol, diabetes, and hypertension. Considering that the choroid is important vascular tissue related with major macula disorders [6–8], the finding that there are genetic effects on CVI could be meaningful.

Several reports investigated the heritability or genetic influence of choroidal thickness (or choroidal volume) [10, 11, 24]. However, there are many factors that influence choroidal thickness, including age, sex, and AXL [25]. In contrast, a population-based study of 345 healthy eyes reported that while choroidal thickness was affected by many ocular and systemic factors, CVI remained relatively unaffected [13]. Additionally, since the choroid is primarily a vascular structure, CVI may be more suitable than the choroidal thickness for assessing the heritability of the choroid. To the best of our knowledge, this is the first report of the heritability of CVI.

In the present study, CVI was found to decrease with increasing age. The association between aging and CVI is still controversial. Some studies reported that CVI decreased with increase in age [26, 27], while other studies reported that CVI remained constant regardless of age [13, 28]. One of the reasons for this difference may be that the measurement method of CVI differs between studies; however, the present study with the largest number of subjects among CVI studies in healthy eyes may provide an important evidence that CVI decreases with age.

CVI was also found to decrease with IOP, and diastolic BP, and with decreasing pulse pressure. Several studies showed an increase in choroidal blood flow by increasing the ocular perfusion pressure [29, 30]. When IOP increases or pulse pressure (systolic BP–diastolic BP) decreases, the ocular perfusion pressure decreases. With the decrease in ocular perfusion pressure, the choroidal blood flow and choroidal vascularity can decrease. However, the relationship between diastolic BP and CVI in this study cannot be explained by the above hypothesis, and it is possible that it is related to autoregulation or is influenced by other factors such as the diurnal variation or time difference between measurements [29, 30].

In addition to these variables, polymorphisms in genetic loci might affect the results. Gene variants, such as rs3793217 (vasoactive intestinal peptide receptor 2; VIPR2) and rs800292 (complement factor H; CFH) have been discovered to be significantly linked with choroidal thickness in a healthy Japanese cohort [24]. CFH has a vasodilatory action when it binds to adrenomedullin, which may promote to dilate the choroidal blood vessels [31]. Additionally, a VIPR2 agonist plays a role in modulating corticosteroid secretion, that may be connected to the mineralocorticoid pathway, in as well as having vasodilatory effects in numerous vascular tissues [32, 33]. Recently, rs2379120 at the GATA5 gene was found to be associated with CVI in patients with chronic central serous chorioretinopathy and may have a role in choroidal vascularity. [34] Further studies will be needed to fully discover the genetic basis of CVI.

The heritabilities of TCA and LA were slightly lower than that of CVI. These results suggest that the proportion of vascular components in the choroid is genetically influenced more than the total choroid or the vascular component itself. Nevertheless, because the explanation for the variation in CVI can be either a change in the number of blood vessels or a change in the diameter of each blood vessel [35], it is not possible to distinguish which of the two is associated with this high heritability.

Although this large healthy twin study has strengths, the investigation also had several limitations. First, only Korean participants were included in this study. Inclusion of various ethnic groups may be more accurate in investigating the heritability of CVI. This needs to be carefully considered when extrapolating these findings to other racial groups. Second, due to the proportion of DZ-MZ in the Korean population, this study’s cohort contained significantly fewer DZ than MZ twins [36, 37]. Nonetheless, given that DZ siblings were treated as equivalent to siblings in the heritability analysis, we made a concerted effort to include a substantial number of extended family members in the study to mitigate the limitations associated with the low count of DZ siblings. Furthermore, the heritability would be greater if the analysis was performed with only MZ twins. Future studies investigating the heritability of CVI using only MZ twins will be needed. Third, the questionnaire used in this investigation did not contain questions about clinical information such as duration of diabetes, history of treatment for hypertension or diabetes, medication use, and usage of steroid that could potentially affect choroidal vascularity; hence, this study could not analyze data by adjusting these factors. Fourth, heritability studies can only reveal information about the genes’ cumulative effects when combined. Thus, further investigations are required to reveal the specific genes which determine CVI.

In conclusion, this investigation shed light on the hereditary basis of choroidal vascularity. The heritability of CVI was high. Moreover, this is the largest population-based study for CVI. Our results may provide a fine insight for the future research on genetics and an understanding of the vascularity of the choroid.

## Abbreviations

AMD: age-related macular degeneration
AXL: axial length
BP: blood pressure
CFH: complement factor H
CVI: Choroidal vascularity index
DZ: dizygotic
EDI: enhanced depth imaging
IOP: intraocular pressure
LA: luminal area
MZ: monozygotic
OCT: optical coherence tomography
TCA: total choroidal area

## References

[1] Hornbeak DM, Young TL. Myopia genetics: a review of current research and emerging trends. Curr Opin Ophthalmol. 2009; 20: 356–362. doi: 10.1097/ICU.0b013e32832f8040 19587595 PMC3736551

[2] Klein RJ, Zeiss C, Chew EY, Tsai JY, Sackler RS, Haynes C, et al. Complement factor H polymorphism in age-related macular degeneration. Science. 2005; 308: 385–389. doi: 10.1126/science.1109557 15761122 PMC1512523

[3] Springelkamp H, Iglesias AI, Mishra A, Hohn R, Wojciechowski R, Khawaja AP, et al. New insights into the genetics of primary open-angle glaucoma based on meta-analyses of intraocular pressure and optic disc characteristics. Hum Mol Genet. 2017; 26: 438–453. doi: 10.1093/hmg/ddw399 28073927 PMC5968632

[4] Klein AP, Suktitipat B, Duggal P, Lee KE, Klein R, Bailey-Wilson JE, et al. Heritability analysis of spherical equivalent, axial length, corneal curvature, and anterior chamber depth in the Beaver Dam Eye Study. Arch Ophthalmol. 2009; 127: 649–655. doi: 10.1001/archophthalmol.2009.61 19433716 PMC2739587

[5] Bloch E, Yonova-Doing E, Jones-Odeh E, Williams KM, Kozareva D, Hammond CJ. Genetic and Environmental Factors Associated With the Ganglion Cell Complex in a Healthy Aging British Cohort. JAMA Ophthalmol. 2017; 135: 31–38. doi: 10.1001/jamaophthalmol.2016.4486 27893004

[6] Cheung CMG, Lee WK, Koizumi H, Dansingani K, Lai TYY, Freund KB. Pachychoroid disease. Eye (Lond). 2019; 33: 14–33. doi: 10.1038/s41433-018-0158-4 29995841 PMC6328576

[7] Siedlecki J, Schworm B, Priglinger SG. The Pachychoroid Disease Spectrum-and the Need for a Uniform Classification System. Ophthalmol Retina. 2019; 3: 1013–1015. doi: 10.1016/j.oret.2019.08.002 31810570

[8] Spaide RF. Age-related choroidal atrophy. Am J Ophthalmol. 2009; 147: 801–810. doi: 10.1016/j.ajo.2008.12.010 19232561

[9] Spaide RF. Disease Expression in Nonexudative Age-Related Macular Degeneration Varies with Choroidal Thickness. Retina. 2018; 38: 708–716. doi: 10.1097/IAE.0000000000001689 28505013

[10] Sardell RJ, Nittala MG, Adams LD, Laux RA, Cooke Bailey JN, Fuzzell D, et al. Heritability of Choroidal Thickness in the Amish. Ophthalmology. 2016; 123: 2537–2544. doi: 10.1016/j.ophtha.2016.09.001 27771146 PMC6613578

[11] Hwang S, Kang M, Ham DI, Kong M. Genetic Influence on Choroidal Volume. Am J Ophthalmol. 2021; 224: 143–149. doi: 10.1016/j.ajo.2020.12.008 33340507

[12] Chhablani J, Barteselli G, Wang H, El-Emam S, Kozak I, Doede AL, et al. Repeatability and reproducibility of manual choroidal volume measurements using enhanced depth imaging optical coherence tomography. Invest Ophthalmol Vis Sci. 2012; 53: 2274–2280. doi: 10.1167/iovs.12-9435 22427584 PMC3995568

[13] Agrawal R, Gupta P, Tan KA, Cheung CM, Wong TY, Cheng CY. Choroidal vascularity index as a measure of vascular status of the choroid: Measurements in healthy eyes from a population-based study. Sci Rep. 2016; 6: 21090. doi: 10.1038/srep21090 26868048 PMC4751574

[14] Agrawal R, Chhablani J, Tan KA, Shah S, Sarvaiya C, Banker A. Choroidal Vascularity Index in Central Serous Chorioretinopathy. Retina. 2016; 36: 1646–1651. doi: 10.1097/IAE.0000000000001040 27124882

[15] Agrawal R, Li LK, Nakhate V, Khandelwal N, Mahendradas P. Choroidal Vascularity Index in Vogt-Koyanagi-Harada Disease: An EDI-OCT Derived Tool for Monitoring Disease Progression. Transl Vis Sci Technol. 2016; 5: 7. doi: 10.1167/tvst.5.4.7 27525196 PMC4970799

[16] Agrawal R, Salman M, Tan KA, Karampelas M, Sim DA, Keane PA, et al. Choroidal Vascularity Index (CVI)—A Novel Optical Coherence Tomography Parameter for Monitoring Patients with Panuveitis? PLoS One. 2016; 11: e0146344. doi: 10.1371/journal.pone.0146344 26751702 PMC4713828

[17] Tan KA, Laude A, Yip V, Loo E, Wong EP, Agrawal R. Choroidal vascularity index—a novel optical coherence tomography parameter for disease monitoring in diabetes mellitus? Acta Ophthalmol. 2016; 94: e612–e616. doi: 10.1111/aos.13044 27151819

[18] Velaga SB, Nittala MG, Vupparaboina KK, Jana S, Chhablani J, Haines J, et al. Choroidal Vascularity Index and Choroidal Thickness in Eyes with Reticular Pseudodrusen. Retina. 2020; 40: 612–617. doi: 10.1097/IAE.0000000000002667 31634322

[19] Sung J, Cho SI, Song YM, Lee K, Choi EY, Ha M, et al. Do we need more twin studies? The Healthy Twin Study, Korea. Int J Epidemiol. 2006; 35: 488–490. doi: 10.1093/ije/dyi294 16423926

[20] Sung J, Cho SI, Lee K, Ha M, Choi EY, Choi JS, et al. Healthy Twin: a twin-family study of Korea—protocols and current status. Twin Res Hum Genet. 2006; 9: 844–848. doi: 10.1375/183242706779462822 17254419

[21] Kong M, Hwang S, Ko H, Song YM, Ham DI. Heritability of Inner Retinal Layer and Outer Retinal Layer Thickness: The Healthy Twin Study. Sci Rep. 2020; 10: 3519. doi: 10.1038/s41598-020-60612-3 32103112 PMC7044332

[22] Kong M, Hwang S, Ko H, Lee GI, Kang M, Sung J, et al. Heritability of macular ganglion cell inner plexiform layer thickness as determined by optical coherence tomography: the Healthy Twin Study. Br J Ophthalmol. 2021; 105: 1011–1015. doi: 10.1136/bjophthalmol-2020-316512 32788326

[23] Hwang S, Kong M, Ko H, Ham DI, Song YM. Genetic influence on macular retinal nerve fibre layer thickness according to retinal subfield. Br J Ophthalmol. 2020; 104: 1448–1452. doi: 10.1136/bjophthalmol-2019-314618 31959589

[24] Hosoda Y, Yoshikawa M, Miyake M, Tabara Y, Ahn J, Woo SJ, et al. CFH and VIPR2 as susceptibility loci in choroidal thickness and pachychoroid disease central serous chorioretinopathy. Proc Natl Acad Sci U S A. 2018; 115: 6261–6266. doi: 10.1073/pnas.1802212115 29844195 PMC6004488

[25] Barteselli G, Chhablani J, El-Emam S, Wang H, Chuang J, Kozak I, et al. Choroidal volume variations with age, axial length, and sex in healthy subjects: a three-dimensional analysis. Ophthalmology. 2012; 119: 2572–2578. doi: 10.1016/j.ophtha.2012.06.065 22921388 PMC3514583

[26] Sonoda S, Sakamoto T, Yamashita T, Uchino E, Kawano H, Yoshihara N, et al. Luminal and stromal areas of choroid determined by binarization method of optical coherence tomographic images. Am J Ophthalmol. 2015; 159: 1123–1131 e1. doi: 10.1016/j.ajo.2015.03.005 25790737

[27] Nivison-Smith L, Khandelwal N, Tong J, Mahajan S, Kalloniatis M, Agrawal R. Normal aging changes in the choroidal angioarchitecture of the macula. Sci Rep. 2020; 10: 10810. doi: 10.1038/s41598-020-67829-2 32616774 PMC7331638

[28] Zhou H, Dai Y, Shi Y, Russell JF, Lyu C, Noorikolouri J, et al. Age-Related Changes in Choroidal Thickness and the Volume of Vessels and Stroma Using Swept-Source OCT and Fully Automated Algorithms. Ophthalmol Retina. 2020; 4: 204–215. doi: 10.1016/j.oret.2019.09.012 32033714 PMC7812781

[29] Riva CE, Titze P, Hero M, Petrig BL. Effect of acute decreases of perfusion pressure on choroidal blood flow in humans. Invest Ophthalmol Vis Sci. 1997; 38: 1752–1760. 9286263

[30] Bogner B, Tockner B, Runge C, Strohmaier C, Trost A, Branka M, et al. The effect of vasopressin on choroidal blood flow, intraocular pressure, and orbital venous pressure in rabbits. Invest Ophthalmol Vis Sci. 2011; 52: 7134–7140. doi: 10.1167/iovs.11-7791 21791588 PMC3207716

[31] Miki A, Kondo N, Yanagisawa S, Bessho H, Honda S, Negi A. Common variants in the complement factor H gene confer genetic susceptibility to central serous chorioretinopathy. Ophthalmology. 2014; 121: 1067–1072. doi: 10.1016/j.ophtha.2013.11.020 24365176

[32] Nussdorfer GG, Malendowicz LK. Role of VIP, PACAP, and related peptides in the regulation of the hypothalamo-pituitary-adrenal axis. Peptides. 1998; 19: 1443–1467. doi: 10.1016/s0196-9781(98)00102-8 9809661

[33] Henning RJ, Sawmiller DR. Vasoactive intestinal peptide: cardiovascular effects. Cardiovasc Res. 2001; 49: 27–37. doi: 10.1016/s0008-6363(00)00229-7 11121793

[34] Kaye RA, Peto T, Hogg R, Griffiths H, Sivaprasad S, Lotery AJ, et al. Choroidal Vascularity in Chronic Central Serous Chorioretinopathy and Its Association with Risk Single-Nucleotide Polymorphisms. Retina. 2024; 44: 837–843. doi: 10.1097/IAE.0000000000004024 38109714 PMC11027981

[35] Agrawal R, Ding J, Sen P, Rousselot A, Chan A, Nivison-Smith L, et al. Exploring choroidal angioarchitecture in health and disease using choroidal vascularity index. Prog Retin Eye Res. 2020; 77: 100829. doi: 10.1016/j.preteyeres.2020.100829 31927136

[36] Hur YM, Kwon JS. Changes in twinning rates in South Korea: 1981–2002. Twin Res Hum Genet. 2005; 8: 76–79. doi: 10.1375/1832427053435445 15836815

[37] Park SH, Lim DO. Trends of twin birth in Korea, 1991–2018. J Health Info Stat. 2019; 44: 427–431.

